# Sensitivity and specificity of the EAT-10 and SDQ-DP in identifying the risk of dysphagia in Parkinson's disease

**DOI:** 10.1055/s-0044-1779055

**Published:** 2024-02-07

**Authors:** Adriana Ponsoni, Flavia Pereira Costa, Vinícius Nagy Soares, Camilla Gabriela Silva Santos, Lucia Figueiredo Mourão

**Affiliations:** 1 Universidade Estadual de Campinas, Faculdade de Ciências Médicas, Departamento de Gerontologia, Campinas SP, Brazil.; 2 Universidade Estadual de Campinas, Faculdade de Ciências Médicas, Departamento de Fonoaudiologia, Campinas SP, Brazil.

**Keywords:** Deglutition, Deglutition Disorders, Parkinson Disease, Respiratory Aspiration, Deglutição, Transtornos de Deglutição, Doença de Parkinson, Aspiração Respiratória

## Abstract

**Background** The early identification of risk for dysphagia in patients with Parkinson's disease (PD) is essential for the prevention of nutritional and pulmonary complications.

**Objective** To analyze the sensitivity and specificity of the Swallowing Disturbance Questionnaire (SDQ-PD) and the Eating Assessment Tool (EAT-10) in identifying dysphagia risk in patients with early and intermediate stages of PD.

**Methods** Twenty-nine patients with PD participated in the study. EAT-10 and SDQ-PD questionnaires were applied, and a videofluoroscopic swallowing study. Dysphagia Outcome and Severity Scale (DOSS) was used to classify the presence and severity of dysphagia, and the Penetration-Aspiration Scale (PAS) was used to identify the presence of penetration/aspiration. In the statistical analysis, the sensitivity and specificity of the risk questionnaires were calculated, as well as positive predictive value, negative predictive value, and accuracy.

**Results** EAT-10 to identify the risk of penetration/aspiration revealed a sensitivity of 71.42% and specificity of 45.45%; in the identification of the presence of dysphagia, the sensitivity was 47.61%, and the specificity was 12.5%. The SDQ-PD questionnaire for risk of penetration/aspiration demonstrated a sensitivity of 28.57%, and a specificity of 68.18%. In terms of identifying the presence of dysphagia, the sensitivity was 20%, while the specificity was 44.44%.

**Conclusion** The SDQ-PD revealed low sensitivity and low specificity to identify the presence of dysphagia and/or penetration/aspiration in patients with early and intermediate stages of PD in this sample. Despite its low specificity, the EAT-10 exhibited good sensitivity in indicating the risk of penetration/aspiration.

## Resumo

**Antecedentes** A identificação precoce de risco para disfagia nos pacientes com doença de Parkinson (DP) é fundamental para a prevenção de complicações nutricionais e pulmonares.

**Objetivo** Analisar a sensibilidade e especificidade dos questionários Swallowing Disturbance Questionnaire (SDQ-PD) e Eating Assessment Tool (EAT-10) para a identificação do risco de disfagia em pacientes com DP nos estágios iniciais e intermediários da doença.

**Métodos** Participaram 29 pacientes com DP. Foi realizado a aplicação dos questionários EAT-10 e SDQ-PD e o exame de videofluoroscopia da deglutição. Para a classificação da presença e gravidade da disfagia foi utilizada a escala Dysphagia Outcome and Severity Scale e, para identificação da presença de penetração/aspiração, a escala Penetration-Aspiration Scale (PAS). Na análise estatística, calcularam-se a sensibilidade e a especificidade dos questionários de risco EAT-10 e SDQ-DP e o valor preditivo positivo, o valor preditivo negativo e a acurácia.

**Resultados** A análise do EAT-10 para identificar o risco de penetração/aspiração revelou sensibilidade de 71.42% e especificidade de 45.45%; para identificar a presença de disfagia, a sensibilidade foi de 47,61% e a especificidade de 12.5%. Em relação ao questionário SDQ-PD, para identificar risco de penetração/aspiração, a sensibilidade foi de 28.57% e a especificidade de 68.18% e, para identificar a presença de disfagia, a sensibilidade foi de 20% e a especificidade de 44.44%.

**Conclusão** O questionário SDQ-PD revelou baixa sensibilidade e baixa especificidade para identificar presença de disfagia e/ou penetração/aspiração em pacientes com DP em estágios iniciais e intermediários para essa amostra. O EAT-10 revelou boa sensibilidade na indicação de risco de penetração/aspiração, apesar de baixa especificidade.

## INTRODUCTION

Dysphagia is a frequent and highly relevant symptom of Parkinson's disease (PD) and is prominent in the advanced stages of the disease. 1 However, changes in swallowing physiology can be detected in the early stages of PD. 2 Instrumental exams, such as the Fiberoptic Endoscopic Evaluation of Swallowing (FESS) and videofluoroscopic swallowing studies (VFSS), make it possible to identify impairments in swallowing physiology in up to 95% of patients with PD. 3

Swallowing screening tools Eating Assessment Tool (EAT-10) and Swallowing Disturbance Questionnaire (SDQ-PD) are widely used by speech-language pathologists and neurologists in the clinical assessment of PD because they are easy to use and provide a quick and inexpensive alternative when specialized professionals and instrumental swallowing tests are unavailable. 4 However, there are discrepancies in the prevalence of dysphagia in PD, with a prevalence of 35% and 85% when using questionnaires and instrumental assessments, respectively. 5 Schlickewei et al., 6 in a study using the EAT-10 questionnaire with 50 patients, did not find a correlation with aspiration/penetration scores, indicating that the EAT-10 failed to identify 38% of penetration/aspiration cases during FESS. In another study, 95% of 119 PD patients did not report swallowing problems but exhibited signs of penetration/aspiration during FEES. 3

Although the use of questionnaires for screening dysphagia risk in patients with PD has been widely discussed, 3 6 7 8 9 there are controversies regarding these instruments because patients with PD often do not report changes in swallowing physiology, 3 5 6 8 10 which can lead to a delayed diagnosis of dysphagia. 11 This delay can worsen symptoms and lead to clinical complications, often resulting in hospitalization for bronchoaspiration pneumonia. 1 2 Thus, we hypothesized that these questionnaires have low accuracy in detecting dysphagia and penetration/aspiration in patients in the early and intermediate stages of PD. We, therefore, aimed to analyze the sensitivity and specificity of the SDQ-PD and EAT-10 questionnaires to identify dysphagia risk comparing to videofluoroscopic swallowing exam in the early and intermediate stages of PD.

## METHODS

### Sample calculation

The sample size calculation was based on a dysphagia prevalence of 36.9% in PD (95% CI: 30.7–43.6%) according to Gong et al. 10 To determine the sample size, the statistical software G*power 3.1.2 was used, analyzing the difference between means with a 95% confidence interval, a significance level of 5%, a margin of error of 5, and an estimated standard deviation of 12.9, as per the prevalence value from the literature (36.9% with a 95% CI: 30.7–43.6%). 10 The resulting sample included 29 patients.

### Study setting and participants

The study was approved by the Ethics Committee of the Researchers' University (91231518.3.0000.5404). Written informed consent was obtained from all the patients. In this cross-sectional study, patients were selected from the Movement Disorders Clinic of Hospital Clínicas at the University of Campinas (UNICAMP). A total of 36 patients were recruited; however, only 29 met the inclusion and exclusion criteria. All assessments in the study were performed during the “on” phase of medication.

The inclusion criteria for participants were as follows:

- neurological diagnosis of PD, according to the Brain Bank of London criteria 12 ;
- classification in the initial and intermediate stages (1 to 3) on the Hoehn & Yahr (H&Y) scale 13 ;
- no cognitive changes on the Scales for Outcomes in Parkinson's disease - Cognition (SCOPA-COG) 14 ;
- no use of benzodiazepines; and
- use of antiparkinsonian medication for at least 30 days before the start of the study.

The exclusion criteria were as follows:

- contraindication to swallowing videofluoroscopy;
- use of tracheostomy or an alternative feeding route;
- previous history of food allergy or allergy to barium sulfate; and
- history of cancer and neurological comorbidities.

Sociodemographic data, including H&Y scales 13 the Unified Parkinson's Disease Rating Scale (UPDRS) total score, motor subscale scores (UPDRS- III), and subscale scores for experiences of daily living (UPDRS-II) question speech and question swallowing. 15 SCOPA-COG, 14 PD duration, age, and sex, were collected from medical records. All the above diagnostic scales for PD were performed by neurologists during consultations at the Movement Disorders Clinic.

### Swallowing assessment

The SDQ-PD ^7^ and EAT-10 16 questionnaires were administered by speech-language pathologists with experience in dysphagia. Although the instruments were self-assessments, the researcher assisted in reading the questions whenever requested by the patients.

### EAT-10 questionnaire

The EAT-10 is a questionnaire that has been translated into Brazilian Portuguese. 17 It consists of 10 questions, and the answers range from “0, no problem” to “4, very large problem”, with the sum ranging from 0 to 40, and a score ≥ 3 meaning that the patient is at risk of dysphagia. 17

### SDQ-PD questionnaires

The SDQ-PD was translated and transculturally adapted into Brazilian Portuguese. 18 This instrument consists of 15 questions, five of which are related to the oral phase of swallowing and 10 of which are related to the pharyngeal phase. In this questionnaire, the responses to 14 questions are measured on a scale of 0 (never) to 3 (very often), and one question is binary (yes or no). A score ≥ 11 indicates a risk of dysphagia. 18

### Videofluoroscopic swallowing study (VFSS)

Speech therapists who had no access to the questionnaire responses conducted the VFSS. The evaluators were experienced in performing the examination and received training and calibration using a Modified Barium Swallow Impairment Profile (MBSImP) protocol. 19 The examination was performed using an X-ray machine (Shimadzu, 120 kV and 800 mA) at the researchers' university.

In the assessment protocol, examinations were divided into two parts. 19 The first part evaluated the lateral view, in which the following consistencies and volumes were classified according to the International Dysphagia Diet Standardization Initiative (IDDSI) 20 : IDDSI 1, fine liquid (5 ml in the spoon, single sip from the glass, and continuous sips); liquid at IDDSI 2 consistency (5 ml from the spoon, single sip from the glass, and continuous sips); thickened liquid at IDDSI 3 consistency (5 ml in the spoon); thickened liquid, consistency IDDSI 4 (5 ml in the spoon); and solid IDDSI 6 (cookie). The second part evaluated the anteroposterior view, in which only the IDDSI 2 (5 ml on the spoon) and IDDSI 4 consistencies (5 ml on the spoon) were evaluated.

For the VFSS analysis, the computer was coupled to the X-ray, and a digital capture was performed using the Pinnacle Studio Video Editing software. All examinations were analyzed by a speech therapist who was “blinded” and certified for analysis using the MBSImP. 19 The frame-by-frame analysis and assignment of numerical codes (magnitude of change) were performed according to a standardized protocol.

The Dysphagia Outcome and Severity Scale (DOSS) 21 was used to classify the presence and severity of dysphagia, with scores ranging from 1 to 7 as follows: (1) severe dysphagia, (2) moderately severe dysphagia, (3) moderate dysphagia, (4) mild to moderate dysphagia, (5) mild dysphagia, (6) functional limits, and (7) normal swallowing.

On the DOSS scale, we considered levels 1–5 as the presence of dysphagia and levels 6–7 as normal swallowing. 21

VFSS examinations were also analyzed in relation to the presence/absence of laryngeal penetration and aspiration using the Penetration-Aspiration Scale (PAS), 22 which assesses the penetration and/or aspiration of food in the airways in a standardized manner. The scale has a classification composed of eight levels: (1) contrast does not penetrate the laryngeal airway invasion; (2) penetration into the larynx above the vocal folds without residue; (3) contrast remains above the vocal folds, with visible residue; (4) contrast visualized in vocal folds with absence of residue; (5) vocal fold contrast with residue; (6) contrast below the vocal folds, can perform cleaning; (7) contrast below the vocal folds without effective cleaning; and (8) silent aspiration.

The PAS was divided into two categories :levels 1-2 and 3-8. 23 Given that levels 1-2 are considered the absence of penetration and/or aspiration, and levels 3-8 indicate the presence of penetration and/or aspiration. 23

### Statistical analysis

SPSS version 20 was used for data analysis. First, the Kolmogorov–Smirnov test was applied to analyze the normality of the data distribution. Subsequently, Student's t-test was performed to compare the mean and standard deviation of the duration of disease, H&Y, 13 UPDRS, 15 and age.

In the statistical analysis of the sensitivity and specificity of the screening instruments (EAT-10 and SDQ-PD), the videofluoroscopic swallowing study is considered the gold standard using the PAS and DOSS scales to quantify the data. The PAS was divided into two categories with dichotomous variables: absence of penetration/aspiration (levels between 1 and 2 were considered the absence of penetration and/or aspiration in the airways); and presence of penetration/aspiration (levels 3 to 8 were considered the presence of penetration and/or aspiration). 23 For statistical analysis of the presence or absence of dysphagia, a dichotomous classification of the DOSS scale 21 was also performed with the following categories: absence of dysphagia (levels 6–7) and presence of dysphagia (levels 1–5). 21

The following statistical analyses were performed:

- analysis of the specificity and sensitivity of the EAT-10 compared with the results of the videofluoroscopic swallowing study (gold standard exam); and
- analysis of the specificity and sensitivity of the SDQ-PD compared with the results of the videofluoroscopic swallowing study. Positive predictive value, negative predictive value, and accuracy were calculated.

## RESULTS

A total of 29 patients were included (eight women and 21 men, with a mean age of 66 ± 14 years). The SDQ-PD, a questionnaire used specifically to screen for dysphagia risk in patients with PD, had a mean of 9.39 ± 6.13, which was below the dysphagia risk score of >11. In the dysarthria and dysphagia subscales of the UPDRS, the mean score for voice-related complaints was 0.20 ± 0.41 and 0.27 ± 0.52 for swallowing-related complaints. The EAT-10 instrument had a mean of 4.24 ± 3.43; that is, the questionnaire identified that the group of participants had a dysphagia risk, considering a score of >3 points as altered ( Table 1 ).

**Table 1:** Clinical and demographic characterization the patients PD (N = 29)

Variables				Min -Max
**Demographic characteristics**	Age		66.24 (±13.98)	(41 - 86)
	Sex	M	21 (72.41%)	
		F	8 (27.58%)	
**Clinical features**	Duration of illness		9.26 (±6.83)	(2 - 23)
	H&Y		2.5 (±0.5)	(1.5 - 3)
	Total UPDRS		37.06 (±13.96)	(15 - 67)
	UPDRS III - total score of motor scale		18.71 (±7.93)	(6 - 29)
	UPDRS II - speech		0.20 (±0.41)	(0 - 1)
	UPDRS II - swallowing		0.27 (±0.52)	(0 - 2)
**Questionnaires**	EAT-10 score		4.24 (±3.43)	(0 - 18)
	EAT-10	At risk	17 (58.62%)	
		No risk	12 (41.38%)	
	SDQ-PD score		9.39 (±6.13)	(0.5 - 23.5)
	SDQ-PD	At risk	9 (31.04%)	
		No risk	20 (68. 96%)	

Abbreviations: F, Females; M, Male; min, minimum; max, maximum; UPDRS, Unified Parkinson's Disease Rating Scale; UPDRS III-total score of motor scale, Unified Parkinson's Disease Rating Scale, motor subscale scores; UPDRS II-speech, Unified Parkinson's Disease Rating Scale, subscale scores for experiences of daily living, question speech; UPDRS II-swallowing, Unified Parkinson's Disease Rating Scale, subscale scores for experiences of daily living, question swallowing; EAT-10, Eating Assessment Tool; SDQ-PD, Swallowing Disturbance Questionnaire; VFSS, Videofluoroscopy of swallowing; H&Y, Hoehn & Yahr.

Note: The variables will be presented as number (%) and mean (± SD).

It is noteworthy that the patients in the present study were in the initial and intermediate stages of PD and 24.15% had already presented with aspiration/penetration (20.70% (PAS 3), and 3.45% (PAS 8)) and 68.98%, the presence of dysphagia [65.51% (DOSS 5) and 3.45% (DOSS 2)], which could not be identified only with the use of questionnaires ( Table 2 ). Out of 20 patients confirmed to have dysphagia through the VFSS (scale DOSS), only four presented with dysphagia risk according to the SDQ-DP. In contrast, for the EAT-10, 11 were identified as having dysphagia risk according to the questionnaire. Regarding the presence of penetration/aspiration, the EAT-10 questionnaire identified five patients at risk of actual penetration/aspiration as seen in the VFSS results. However, the SDQ-PD identified only two patients at-risk patients who had penetration/aspiration confirmed through VFSS ( Table 3 ).

**Table 2:** Videofluoroscopy of swallowing and PAS scales and DOSS scales

Videofluoroscopy of swallowing		Levels	
PAS	Absence of penetration/aspiration	1	7 (24.13%)
		2	15 (51.72%)
	Presence of penetration/aspiration	3	6 (20.70%)
		4 - 7	0
		8	1 (3.45%)
DOSS	Functional/normal swallowing	7	1 (3.45%)
		6	8 (27.59%)
	Dysphagia	5	19(65.51%)
		4 - 3	0
		2	1 (3.45%)
		1	0

Abbreviations: DOSS, Dysphagia Outcome and Severity Scale; M, Men; PAS, Penetration-Aspiration Scale; SD, Standard deviation; W, Women.

Notes: PAS: level 1 - contrast does not penetrate the laryngeal airway invasion; level 2 - penetration into the larynx above the vocal folds without residue; level 3 - contrast remains above the vocal folds, with visible residue; level 4 - contrast visualized in vocal folds with absence of residue; level 5 - vocal fold contrast with residue; level 6 - contrast below the vocal folds, can perform cleaning; level 7 - contrast below the vocal folds without effective cleaning; and level 8 - silent aspiration; DOSS : level 1 - severe dysphagia, 2 - moderately severe dysphagia, 3 - moderate dysphagia, 4 - mild to moderate dysphagia, 5 - mild dysphagia, 6 - functional limits, and 7 - normal swallowing.

**Table 3:** Analysis the dysphagia risk of questionnaires EAT -10 and SDQ-PD (N = 29) and the presence of penetration/aspiration and dysphagia in the VFSS

SDQ- PD questionnaire		VFSS			
		PAS N = 29		DOSS N = 29	
		P/A	Absence in the P/A	Dysphagia	Absence in dysphagia
**Dysphagia risk**	At risk	2	7	4	5
	No risk	5	15	16	4
**EAT-10 questionnaire**	At risk	5	12	11	7
	No risk	2	10	9	2

Abbreviations: DOSS, Dysphagia Outcome and Severity Scale; EAT-10, Eating Assessment Tool; P/A, Penetration/aspiration; PAS, Penetration-Aspiration Scale; SDQ-PD, Swallowing Disturbance Questionnaire; VFSS, Videofluoroscopy of swallowing.

The SDQ-PD demonstrated a low sensitivity (28.57%) in identifying the presence of penetration/aspiration by PAS. The specificity of the SDQ-PD questionnaire was 68.18% compared with PAS. Furthermore, when comparing the SDQ-PD questionnaire with the DOSS scale, it was found to have a low sensitivity of 20% and a low specificity of 44.44%. This indicates that the SDQ-PD may not be as effective as the DOSS in correctly identifying dysphagia risk in some participants ( Table 4 ).

**Table 4:** Comparison of sensitivity and specificity with the videofluoroscopy swallowing exam using the PAS and DOSS

Parameters (%s)	EAT-10		SDQ-PD	
	PAS	DOSS	PAS	DOSS
Sensitivity	71.42%	47.61%	28.57%	20%
Specificity	45.45%	12.5%	68.18%	44.44%
PPV	29.41%	58.82%	22.22%	44.44%
NPV	83.33%	8.3%	75%	20%
Accuracy	51.72%	37.93%	58.62%	27.58%

Abbreviations: DOSS, Dysphagia Outcome and Severity Scale; EAT-10, Eating Assessment Tool; NPV, Negative predictive value; PAS, Penetration-Aspiration Scale; PPV, Positive predictive value; SDQ-PD, Swallowing Disturbance Questionnaire.

In the analysis of the sensitivity and specificity of EAT-10 for the indication of risk for penetration/aspiration (determined by PAS), a high sensitivity (71.42%) but low specificity (45.45%), was observed. However, for the identification of the presence or absence of dysphagia, the EAT-10 had low sensitivity (47.61%) and specificity (12.5%) ( Table 4 ). In the present study, because of low sensitivity and specificity, it was not possible to generate a receiver operating characteristic (ROC) curve.

## DISCUSSION

Our hypotheses were confirmed, and the questionnaires did not seem to be sufficient to detect dysphagia risk. However, the EAT-10 questionnaire, despite not being specific to PD, showed high sensitivity in identifying penetration/aspiration in patients in the early or intermediate stages of PD.

Symptoms of dysphagia may be less common in PD. 1 2 One explanation could be the impairment of the glossopharyngeal and vagus nerves during sensation, even in the early stages of the disease. 24 25 Another aspect that may be related is that dysphagia symptoms are more evident for family members and caregivers in the advanced stages of the disease, 26 with the presence of several changes, such as decreased tongue strength 27 and laryngeal penetration and aspiration, 3 4 which interfere with the safe ingestion of medication and food. 11

When analyzing the sensitivity and specificity of EAT-10 in the context of identifying penetration/aspiration (PAS), the sensitivity was high (71.42%), but the specificity and accuracy were low. Low sensitivity (47.61%) and low specificity (12.5%) were found for the detection of dysphagia risk. The data of the present study confirm the results of a previous study that aimed to evaluate the ability of EAT-10 to predict the risk of penetration/aspiration in patients with PD. 6

Our findings differ from those of studies conducted with other diseases, such as chronic obstructive pulmonary disease and amyotrophic lateral sclerosis, that showed a correlation between EAT-10 scores and the presence of dysphagia during FEES. 28 29 This might be related to the disease's pathology, with preservation of sensory pathways, in which patients are able to report changes in swallowing from the early stages of the disease.

Studies have reported that the EAT-10 has diagnostic accuracy; however, there is no consensus regarding the cutoff score. Some studies 30 31 point out that a cutoff score of 2 would demonstrate greater sensitivity of the EAT-10, but would also increase the rate of diagnostic errors. Belafsky et al. 16 the authors of the EAT-10, and other researchers have suggested the use of a cutoff score of 3 because it presents a better balance between sensitivity and specificity, and greater diagnostic accuracy. 16 32 In a meta-analysis performed by Zhang et al., 32 a cutoff score between 2 and 3 was suggested.

The SDQ-PD demonstrated low sensitivity and specificity, with a cutoff score >11 when considering penetration/aspiration and the presence of dysphagia. These results differ from the literature, which suggests that the SDQ-PD is a questionnaire capable of identifying dysphagia risk in the PD population with good sensitivity (80.5%) and specificity (81.3%). 7 In contrast, we revealed a sensitivity of 28.57% and specificity of 68.18% in a very similar sample to the study conducted by Manor et al. 7 However, it is important to note that Manor et al. 7 used FEES as the standard evaluation and did not use a scale to assess the presence of penetration/aspiration. It is essential to highlight that the presence of penetration/aspiration during swallowing without residue (PAS 2, 4, and 6) was not identified by FEES; however, VFSS provided greater accuracy in detecting these occurrences. 2 23

Another important factor concerning the SDQ-PD is that, in Brazil, the questionnaire underwent translation and cross-cultural adaptation. 18 However, this has not been validated for Brazilian population. In another study conducted among native Portuguese speakers, the SDQ-DP was translated and adapted across cultures. However, the authors validated only the SDQ-PD construct. 33

Our patients reported few complaints related to swallowing and voice in the UPDRS subscales, similar to a previous study by Nienstedt et al. 3 which highlighted that patients with PD may not respond to or notice changes in swallowing due to the lack of more focused questions on this topic. Another validated questionnaire for dysphagia screening in PD is the Munich Dysphagia Test – Parkinson's Disease 34 ; however, its use is extensive and time-consuming and is not used in routine neurological assessment. 34 In a study conducted by Buhmann et al, 35 the Monique test was found to be ineffective in detecting aspiration risk and laryngeal penetration.

Studies have also highlighted the need for new questionnaires to help identify the risk of dysphagia in patients with PD, as there are significant differences in the prevalence of dysphagia between studies using instrumental assessments and those relying solely on questionnaires 5 36 Cosentino et al. 36 attempted to establish a consensus on the use of evaluations and screenings in patients with PD, and found that few instruments were submitted for cross-cultural translation and validation, which may have affected the present results. 36 They also highlighted the need to provide accurate screening tools for the risk of dysphagia in PD to neurologists, particularly for patients in the early clinical stages, so that these patients can be referred early for clinical and instrumental evaluation of swallowing. 36

Another possibility is to develop multicenter studies with different cutoff scores for the SDQ-PD based on the disease stage. This approach could increase the sensitivity and specificity of the test in patients with early- and intermediate-stage PD. We demonstrated that the EAT-10 is a good instrument to identify signs of aspiration and penetration and can be used in clinical practice as a screening tool for dysphagia risk in patients with PD.

The Swallowing Quality of Life Questionnaire can be used in patients with dysphagia. 37 38 This instrument specifically assesses the impact of swallowing changes on the quality of life and can be used by a multidisciplinary team to assess how changes in swallowing physiology affect patients.

Although it is not possible to request a VFSS for early diagnosis of all patients with PD in the Brazilian health system, it is important to perform an accurate clinical assessment. 1 The speech therapist is a qualified professional who performs clinical swallowing evaluations. 39 We used the VFSS, which is the gold standard for instrumental evaluation in detecting dysphagia, 2 11 and found that questionnaires alone do not demonstrate the risk of dysphagia like an instrumental examination. 36 In the absence of instrumental examinations, it is important to actively search for patients with PD in the early and intermediate stages. In addition, discussions within a multidisciplinary team are crucial to ensure that patients are promptly referred for dysphagia evaluation and treatment.

Our study had some limitations, such as the lack of distribution of the different levels of dysphagia scales used, the PAS, and the DOSS.

In conclusion, compared with the VFSS, the SDQ-PD did not show sufficient sensitivity and specificity in detecting penetration/aspiration and dysphagia in patients with PD in the early and middle stages. In contrast, the EAT-10 showed high sensitivity in identifying the risk of penetration/aspiration, although this was with low specificity. However, the EAT-10 did not prove to be a good tool to indicate the presence of dysphagia.
